# Automated Clinical Dosimetry Planning of Dense Lattice Radiation Therapy

**DOI:** 10.3390/cancers17122048

**Published:** 2025-06-19

**Authors:** David Macias-Verde, Javier Burgos-Burgos, Pedro C. Lara

**Affiliations:** 1 Department of Clinical Sciences, Universidad de Las Palmas de Gran Canaria (ULPGC), Juan de Quesada, 30, 35001 Las Palmas de Gran Canaria, Spain; david.macias@hospitalessanroque.com; 2 Oncology Department, Centro Oncologico Integral Canario (COIC), Hospitales Universitarios San Roque (HUSR), Dolores dela Rocha 5, 35001 Las Palmas de Gran Canaria, Spain; javier.burgos@hospitalessanroque.com; 3 Department of Medicine, Universidad Fernando Pessoa Canarias (UFPC), Mendoza, 6, 35006 Santa Maria de Guia, Spain; 4 Instituto Canario de Investigacion del Cancer (ICIC), Avenida de la Trinidad, 61, 38204 Santa Cruz de Tenerife, Spain

**Keywords:** lattice radiation radiotherapy, LRT, SFRT, fractionated, dense

## Abstract

Background: Patients bearing large-volume, bulky primary or relapsed tumors, are usually referred to palliative low-dose radiotherapy with very poor results. Lattice Radiation Therapy (LRT) is able to produce a high number of high-dose foci or vortexes (multiple SBRT treatments), separated by low-dose zones (valleys). Treatment planning on vortex placing, valley definition, and dose administered depends on individual decisions of the treating team. The aim of our study is to assess for the first time the possibility of a dense fractionated LRT within the target volume. Methods: A total of 22 treatments in 20 patients were performed in the frame of a prospective observational study of fractionated LRT ongoing in our institution. According to our aim of achieving dense LRT, no GTV contraction was considered to create the LRTV (GTV is equal to LRTV). The vortexes were segmented as 1 cm diameter at a 1.5 cm vortex-to-vortex distance. Dose prescription to the vortexes per fraction was 12 Gy. Results: The vortex/LRTV ratio was 7.38 ± 2.13% (3.4–10.40%, median 7.60%). Mean dose to the vortex volume was 11.90 ± 0.09 Gy (11.70–12.10 Gy, median 11.90 Gy). Mean dose administered to the valley volume was 8.29 ± 0.70 (7.05–9.51 Gy, median 8.29 Gy). Valley/vortex (peak) dose ratio (VPDR) was 69.40 ± 6.02% (59.00–79.80%, median 69.70%). The mean peripheral tumor dose was 5.11 ± 0.8710 Gy (3.16–6.78 Gy, median 5.18 Gy). Conclusions: Our dense LRT schedule fulfilled most of the recommended guidelines for LRT, increasing the high dose points without risking the dose to the surrounding tissues. Further analysis of feasibility and safety are needed to secure the clinical relevance of our proposed protocol.

## Simple Summary

Patients bearing large-volume tumors, are usually referred to palliative low-dose radiotherapy with very poor results. Lattice Radiation Therapy (LRT) is able to produce a high number of high-dose foci or vortexes (multiple SBRT treatments), separated by low-dose zones (valleys). The aim of our study is to assess for the first time the possibility of a dense fractionated LRT within the target volume. A total of 22 treatments in 20 patients were performed. The vortexes were segmented as 1 cm diameter at a 1.5 cm vortex-to-vortex distance. Dose prescription to the vortexes per fraction was 12 Gy. The vortex/LRTV ratio was 7.38 ± 2.13% (3.4–10.40%, median 7.60%). Mean dose to the vortex volume was 11.90 ± 0.09 Gy (11.70–12.10 Gy, median 11.90 Gy). Mean dose administered to the valley volume was 8.29 ± 0.70 (7.05–9.51 Gy, median 8.29 Gy). Our dense LRT schedule fulfilled most of the recommended guidelines for LRT, increasing the high dose points without risking the dose to the surrounding tissues.

## 1. Introduction

External Beam Radiotherapy (EBRT) with a curative tumor intent is usually administered in total doses 60–70 Gy at 2 Gy per fraction to the whole tumor area. In contrast, Stereotactic Ablative Radiotherapy (SABR) allows for high Biological Effective Doses of radiation (>100 Gy BED) in a highly conformal manner. Radiation is administered in few fractions (1–5) at high dose per fraction to localized targets to ablate the tumor, increasing local control in small metastatic/primary tumors either in the brain (stereotactic radiosurgery, SRS) or in the body (stereotactic body radiotherapy, SBRT). The low rates of toxic effects reported are very relevant, given the necessity of increasing the quality of life of patients with reduced life expectancy [1,2].

In this SRS/SBRT approach, planning was devoted to achieving a uniform radiation dose to the entire tumor [1,2]. This Stereotactic Ablative Radiotherapy induces blood vessel damage and subsequent necrotic cell death [3], not to mention the potential immune mediated effect either in the short (bystander effect) or long (abscopal effect) ranks [4,5]. Local control rates account for over 90% of the cases [1,2], and the prospect of combination with systemic immunotherapy makes Stereotactic Ablative Radiotherapy a cornerstone treatment for cancer [6]. Stereotactic Ablative Radiotherapy is usually indicated for oligometastatic cancer (1–5 lesions) and primary small tumors. Tumor size to treat is limited by tolerance of surrounding organs at risk (OARs) [1,2]

Unfortunately, some patients bear bulky primary or relapsed tumors that are not amenable to surgical removal or responsive to systemic therapies. Most of these cases are referred to palliative low-dose radiotherapy with very poor results [7,8]. Although it is difficult to find a clear definition of bulky tumors through the radiation literature, in the seminal paper of Mohiuddin M et al. [9], the largest tumors of 8 cm diameter were considered bulky, according to the previous publications that showed a direct relation between tumor volume a radiation response [10]. Indeed, bulky tumors’ radioresistance would be explained by the low oxygen levels within poor vascularized tumor regions and the high tumor cell heterogenicity displaying different sensitivity to treatment [8].

Furthermore, most of these bulky tumors show an immunosuppressive microenvironment, which hinders the immune system’s ability to kill cancer cells. In this situation, combinations with chemotherapy face limitations due to poor drug penetration into the tumor and hypoxia resistance mechanisms [11]. Immune Checkpoint Inhibitors (ICIs) are not useful due to the immunosuppressive environment, with poor tumor PD-L1 expression, commonly defined as cold tumors [12]. Expression of PD-L1, a protein found on tumor cells and some immune system cells, plays a key role in cancer immunotherapy. By binding to PD-L1 on T cells, PD-L1 can inhibit the immune response, allowing cancer cells to evade the immune system’s attack.

Therefore, a different approach from conventionally fractionated radiotherapy should be considered to improve radiation responses in these radioresistant tumors. On the other hand, SABR is limited to small-volume tumors and would be of limited use in these large tumors.

Spatially fractionated radiotherapy (SFRT) provides innovative strategies to overcome tumor resistance to radiotherapy [13]. In this context, Lattice Radiation Therapy (LRT) is envisaged as a novel way to produce a high number of high-dose foci or vortexes, like multiple SABR treatments, separated by zones that received lower doses or valleys [14]. Characteristically, the spatial arrangement of high-dose vortexes follows a three-dimensional pattern, individually customized based on the tumor’s size, shape, and location. These multiple SABR treatments within the tumor volume would allow for a shrinkage of bulky tumors due to fast tumor necrotic cell death [15]. Furthermore, this initial tumor shrinkage may help the effectiveness of other cancer treatments such as chemotherapy, immunotherapy, or standard External Beam Radiation Therapy (EBRT). Proton and heavy ion therapies allow for more precise targeting of tumors while protecting normal tissue, but limits in cost and availability would make lattice radiotherapy a cost-efficient alternative for treating bulky radioresistant tumors [15]. Lattice radiotherapy has shown to obtain a high rate of tumor responses with an excellent safety profile [16,17].

Treatment planning on vortex (peak doses) and valley (dwell doses between peaks) designs are still not fully standardized, as vortex placing, valley definition, and dose administered depends on individual decisions of the treating team [18]. Recommendations from Wu et al. [19] for LRT planning include the following: (a) vortexes should be restricted to remain entirely within the boundaries of the GTV, (b) vortex diameters of 0.5–1.5 cm adopted as the nominal size, (c) vortexes’ center-to-center distance should be 2–5 cm and, finally, (d) the volumetric fraction of the GTV occupied by vortexes should not be greater than 10%.

An inward margin of 1 to 2 cm is also recommended to ensure that the vortexes remain safe from organs at risk (OARs). Regarding the aim of the treatment (palliative debulking or immune stimulant), the prescribed dose at the vortexes may range from 10 to 20 Gy, while the valleys should receive around 5 Gy, devoted to achieving a significant final dose difference between the peaks and valleys, ranging between 50% and 80%. Peripheral tumor dose was suggested to remain below 2 to 5 Gy. Unless vortex volume and LRT volume are well defined, the valley volume definition has no clear recommendations [14,15,19].

Lattice treatment aiming to reduce tumor volume (debulking) as a boost planned in combination with conventional radiotherapy and systemic therapy in radiorresistant bulky tumors accounts for most of the publications [15,16,17,20,21]. Fractionated lattice in doses of around 12 Gy in several fractions is commonly recommended in this situation [15,20,21].

According to these previous considerations, when heading for a palliative debulking LRT, we should be able to produce the highest number of high-dose vortexes within the tumor volume, while keeping low-dose regions under safe conditions for normal tissues and OARs. In fact, any additional dose safely delivered to the tumor would result in an increased tumor cell death. This additional dose is achieved by the increased number of vortexes within the tumor volume (which we refer to as dense LRT). As previously described, fractionation for palliative debulking treatment is commonly administered with fractionated LRT at 12 Gy doses [15,20,21]. Secondly, as vortex and valley definition and dose administered depend on individual decisions [18], a new way to reduce the variations in vortex placing within the tumor volume would be of interest. In this context, automated placing of the vortexes would allow for increased reproducibility of the treatment and optimize tumor coverage. Treatment planning on vortex (peak doses) and valley (dwell doses between peaks) designs are still not fully standardized. Therefore, it would be of interest to standardize automated vortex placing and, at the same time, increase the number of vortexes within the tumor volume (dense LRT).

The aim of our study is to assess for the first time the possibility of an automated dense fractionated LRT and the fulfillment of LRT recommendations on volumes and dose distribution within the target volume.

## 2. Materials and Methods

A total of 22 treatments in 20 patients were performed in the framework of an observational prospective study of fractionated LRT ongoing in our institution. Patient inclusion criteria were as follows: age over 18 years, ECOG 0-2, bulky primary or relapsed tumors >8 cm in the largest diameter, tumors not amenable of curative local treatment with surgery or standard radiotherapy, and systemic therapy refractory/unfit tumors. Exclusion criteria were as follows: ECOG 3, candidates for first-line systemic therapy, and tumors amenable of surgery or conventional radiotherapy. After the CT simulation scan (Discovery IQ PETCT (GE Medical Systems), the delineation of the organs at risk (OAR) and the gross tumor volume (GTV) were segmented by a radiation oncologist on the 3D planning computed tomography (CT) image tool after registration of patient’s available MRI/PET scans. The CT scan slice thickness and the CT pixel image resolution were 3 mm and 512 × 512, respectively.

**Figure 1:**
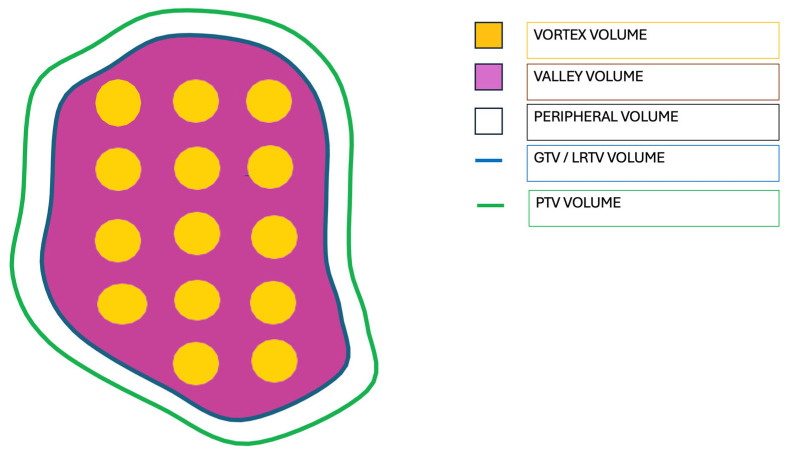
Graphical display of volume definitions of our dense LRT protocol.

According to our aim of achieving dense LRT, no GTV contraction was considered to create the LRTV (GTV is equal to LRTV). Valley volume was defined as the LRTV volume out of the vortex volume. The PTV was defined as the GTV expanded by 10 mm, always considering patient’s individual anatomy and location of organ at risk. The peripheral dose was estimated just outside the LRTV, that is, the volume of the PTV not included in the LRTV (Figure 1).

The vortexes were segmented as 1 cm diameter 3D spheres and placed at the center using the grid tool of the contouring application of the Varian Aria software (Varian Medical Systems, v18.0). A 1.5 cm vortex-to-vortex grid was superimposed on the patient’s CT slices in the three planes—axial, sagittal, and coronal—and a static 3D brush was applied to draw the spheres automatically by clicking only on the chosen vortexes of the grid within the LRTV.

**Figure 2:**
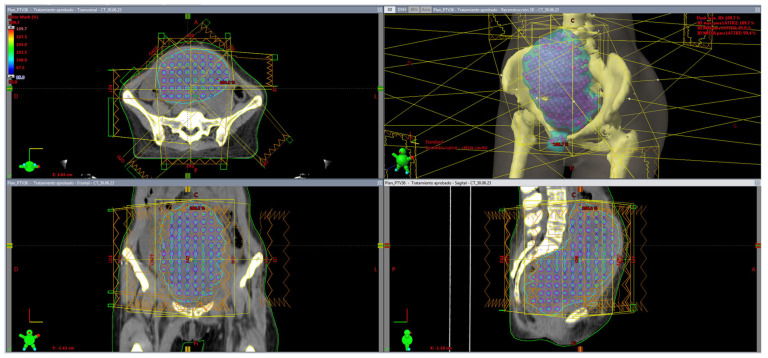
Case #11. Eclipse TPS (Varian Medical Systems) 4—view dosimetry result of 95% dose in color wash. The patient is lying subito supine, feet first, during simulation and treatment. Lattice vortexes are redistributed in the volume.

By positioning only the center on the vertices of the grid, the software will segment spheres along the three planes—axial, coronal, and sagittal (Figure 2). We continue filling the target volume in all its dimensions with equally spaced spheres, always respecting the edge of the segmented target volume. Preselecting a semitransparent texture for the target volume, we check in the 3D contouring window whether the distribution of spheres is uniform in the three axes or not, as we go forward along the total target volume. Organs at risk (OARs) were segmented previously to the vortex placement. However, in some cases, when the vortexes fall within or very close to critical healthy structures or even those segmented by the physician but susceptible of not being suitable to receive very high doses (bone, lung, etc.), spheres in such locations are deleted, thus preserving SFRT dose-gradients as qualitatively evaluated by a physicist and physician at the time of plan review.

As previously described, palliative debulking LRT is recommended in doses of around 12 Gy in several fractions [15]. Dose prescription for all patients included in the study was 12 Gy. We followed the guidelines already available for planning LRT [19]: (a) Dose to the vortexes was prescribed as 95% vortex volume receiving the 95% of the prescribed dose; (b) dose to the valleys was intended to be 5 Gy and have a valley/vortex dose ratio of 50–80%; (c) dose to the OARS should accomplish the Timmermans constraints for SBRT [22] and its peripheral dose should be 2–5 Gy.

Once the segmentation and dose–volume objectives are determined, inverse intensity modulated planning (IMRT) is the next step. The current treatment planning systems (TPS) optimization algorithms are not tailored to LRT. Therefore, multiple iterations are needed to fulfill the planning guidelines and obtain the desired LRT dose distribution. Beam arrangement included a box and/or added oblique fields. All IMRT plans were created in the Varian Eclipse TPS treatment planning system (Varian Eclipse v.15.6 and v.18.), using its Photon Optimizer (PO) for inverse optimization and the Analytical Anisotropic Algorithm (AAA) for dose calculation. We defined an optimizer gradient to normal tissue and an optimizer reduction factor to normal tissue of 1 mm and 0.9, respectively. The Shape Controller Moderate was used for optimizer aperture and Mode On for optimizer convergence. Plans were delivered on a Varian Clinac Unique with Millenium 120.

When performing the clinical dosimetry, we used 6 MV X-ray beams released on the patient with the IMRT Sliding Windows technique. Verification of clinical dosimetry was performed with the electronic portal dosimetry imaging device (EPID: AS-1000) incorporated in the LINAC. Gamma verification dosimetry was 97% of the points, which should fulfill the criteria of 3%/3 mm tolerance gamma analysis.

A detailed numerical and graphical analysis of the dose distribution in the peaks and valleys was performed using the MATLAB software (short for MATrix LABoratory, version: 9.14.0 (R2024b), Natick, MA, USA: The MathWorks Inc.; 2023). The DICOM 2D dose planes extracted from Eclipse has been analyzed using the software for mathematical modeling due to its powerful built-in functions for matrix operations, numerical analysis, and visualization. Detailed study of each planned dose distribution was compared to eclipse available dose distribution data.

The statistical analysis has been performed using the Jamovi Project software (2024, Version 532.6, retrieved from https://www.jamovi.org).

## 3. Results

The 22 cases identified by their diagnosis and by the volume and dose information on vortexes, valleys, LRTV, and periphery, as well as their corresponding percentage ratios, as obtained from Eclipse TPS, are shown in Table 1. As per protocol, all patients were included in a palliative debulking lattice radiotherapy fraction of 12 Gy. Only 3/22 had previously received radiotherapy in the treated area (one limb sarcoma case #3, a tongue carcinoma case #5, and an oral cavity carcinoma case #8)

The LRTV in our study was 415 ± 435 cc (45.40 cc–1863.00 cc, median 326 cc). The vortex volume was 32.60 ± 40.7 cc (1.75 cc–182.00 cc, median 22 cc). The valley volume was defined as the LRTV/GTV out of the vortex volume (mean: 380.00 ± 396.00 cc) (38.90 cc–1674.00 cc, median 296.00 cc). The peripheral volume was 395 ± 229.00 cc (88.80–975.00 cc, median 369.00 cc). Vortex volume-to-LRTV ratio was 7.38 ± 2.13% (3.45–10.40%, median 7.60%).

**Figure 3:**
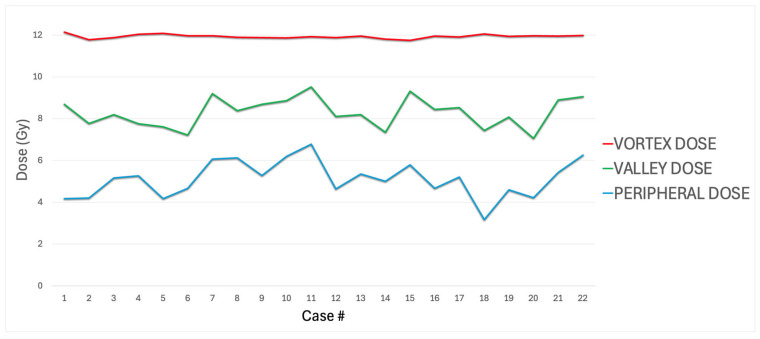
Vortex, valley, and peripheral doses in the 22 treatments.

Treatment was administered by a median of 6.00 (range 3.00–9.00) fixed angles of sliding windows inverse IMRT with a mean monitor unit of 12,327 ± 5154 MU (4197–22,349 MU, median 12,357 MU). Mean dose to the LRTV was 8.56 ± 0.7030 (9.75–7.41 Gy, median 8.59 Gy). Mean dose to the vortex volume was 11.90 ± 0.0946 Gy (11.70–12.10 Gy, median 11.90 Gy). Mean dose administered to the valley volume was 8.29 ± 0.705 (7.05–9.51 Gy, median 8.29 Gy). Valley-to-vortex (peak) dose ratio (VPDR) was 69.40 ± 6.02% (59.00–79.80%, median 69.70%). The mean peripheral tumor dose (out of LRTV/GTV) was 5.11 ± 0.8710 Gy (3.16–6.78 Gy, median 5.18 Gy) (Table 1, Figure 3).

We must realize that LRTV is the sum of vortex volume plus valley volume. Those three factors are, therefore, closely related and followed a normal distribution. Vortex, valleys, and peripheral doses followed a non-normal distribution.

We performed a T test for analyzing if the vortex dose is statistically different from the valley dose and if both are different from the peripheral dose. All three doses were statistically different (*p* < 0.0001). We also performed a correlation analysis of the LRTV, vortex volume, and valley volume with doses at the vortex, valley, and peripheral regions. The peripheral dose was directly statistically related to LRTV (*p* = 0.049), vortex volume (*p* = 0.003), and valley volume (*p* = 0.046). Neither vortex nor valley doses were related to treatment volumes.

Dose to the OARs accomplishes the Timmermans constraints for SBRT in all patients Supplementary File S1. DVH ORGANS AT RISK).

**Figure 4:**
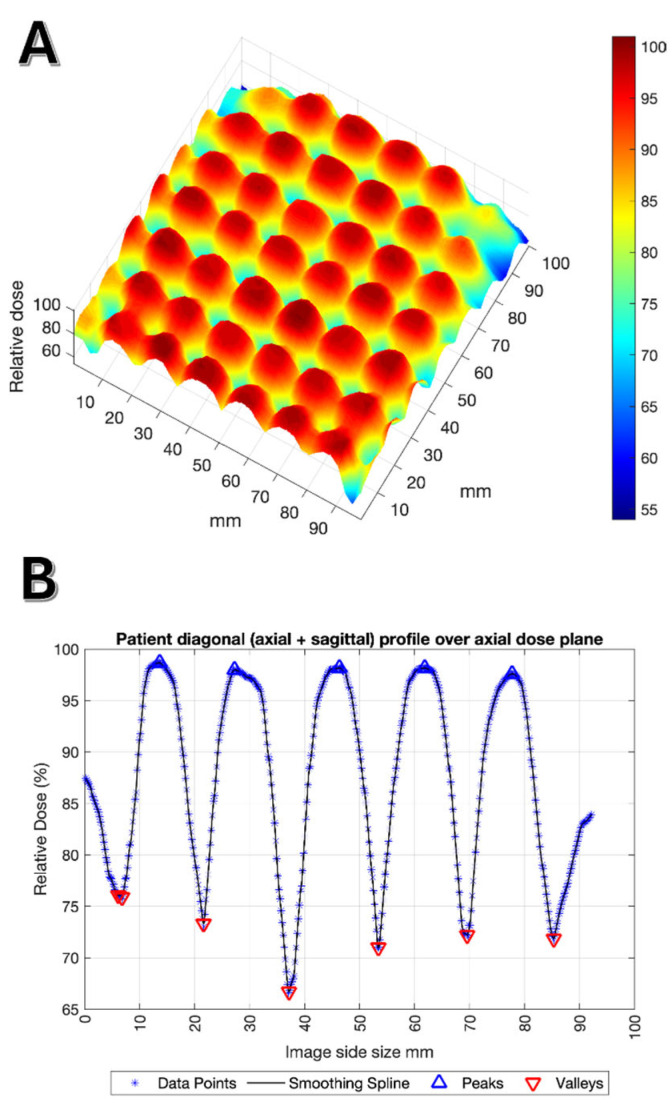
Case #11 out of 22: (**A**) An axial 2D dose plane extracted from Varian Eclipse and plotted as a 3D image surface map using MATLAB; (**B**) relative dose profiles. Data treated with MATLAB version: 9.14.0 (R2024b), Natick, MA, USA: The MathWorks Inc.; 2023.

We analyzed the dose distribution using the MATLAB software The MathWorks, Inc. MATLAB, (Version R2024b). As described in Section 2, DICOM 2D dose planes extracted from Eclipse were analyzed by MATLAB. Figure 4A shows a 3D graph of dose distribution-conforming vortexes and valleys, and Figure 4B shows the section analysis of peaks and valleys in a case study. To interpolate the set of data points along the 2D image over a diagonal profile passing through vortexes (peaks) and valleys to a piecewise polynomial function, a spline fitting in each spatial direction of the dose plane was performed to construct a smooth curve that passes through or near the data points while maintaining a high degree of smoothness (Supplementary File S2. MATLAB SCRIPT).

## 4. Discussion

SFRT is gaining acceptation within the radiation oncology community for the treatment of locally advanced/bulky tumors [23]. Lattice Radiotherapy is one of the treatment options included in the SFRT field [24,25,26,27]. LRT aims to give very high doses to a reduced tumor volume in the form of vortexes, allowing for as low as possible doses in between such vortexes (valleys) and maintain the dose out of the tumor as expected in conventionally fractionated radiotherapy [14,19,25,28]. Placing the vortexes in an automated manner has been recently studied [29,30,31].

We present here for the first time a novel LRT arrangement, where vortexes are densely covering the LRTV/GTV (7.38%). This vortex volume to LRTV/GTV ratio is within the boundaries of the recommended 1–10% ratio, albeit 3 to 4 times higher than the published data [17,18,20]. The vortex-to-LRT volume ratio ranged from 0.5 to 4% in the study from Studer et al. [17], 2% in the study of Wu et al. [19], and 1.9–4.3 in the study of Duriseti et al. [20]. This dense LRT (increased number of vortexes within the tumor volume) would result in higher debulking tumoral doses. This higher vortex-to-LR ratio TV/GTV ratio than those published is explained by the following: (a) our decision to include the whole GTV as LRTV without the pre-planned 1 cm subtraction; (b) we used 1 cm diameter vortex but with a slightly shorter distance between vortexes (1.5 cm from center-to-center). This separation also results in a 2 cm wide distance (center-to-center) in the diagonal axis.

As stated above, we consider that for LRT, palliative debulking treatment, as much as the high-dose vortexes (SBRT point), would be administered to achieve the largest tumor reduction and shrinkage. In any oncological treatment of these bulky, unsuitable tumors, fast tumor volume reduction would allow for increased options of concomitant systemic, radio, or surgical treatments. Furthermore, resistance due to hypoxia and genetic heterogenicity would be better overcome by 12 Gy doses per fraction in a high number of vortexes. The proposed array is within the boundaries of the recommended ratios.

Following our dense vortex array, as we do not perform any preplanning subtraction from the GTV to create the LRTV (LRTV = GTV), the vortexes close to OARs or limiting dose structures were adequately suppressed. Our results confirm that it is possible to cover a larger LRTV with high-dose vortexes (7.38%) than in other trials [19] and that these vortexes receive almost 100% of the prescribed dose (mean/median: 11.90 Gy).

On the other hand, we clearly state that all LRTV not included in the vortex volume should be considered valley volume. Valley volumes and doses were not so clearly defined as vortex volumes and doses [18]. All proposed definitions try to present the valley volume as the region receiving the lowest doses, which may lead to erroneous expectations when assessing the dosimetry results in daily clinical practice [14,19]. As described in Table 1, the LRTV is the sum of vortexes’ and valleys’ volumes. We argue that this is a real, simple way to describe the tumor tissue out of the vortexes and how the dose is distributed within this volume.

We were able to achieve an expected valley-to-vortex volume dose ratio of 69.40 ± 6.02% (59.00–79.80%, median 69.70%) that is clearly within the boundaries of the recommended 50.00–80.00% [19] ratios. It has been stated that when LRT is aimed at a debulking treatment in combination with other treatments, the low dose in the valleys is not so relevant as in those immune-revigorization LRT treatments [15]. In fact, preservation of immune cell populations within the valleys is crucial to obtaining the immune response expected by this kind of immune LRT. In the case of debulking LRT, planning efforts are committed to preserving the patients from severe toxicity [15]. In our study, the very large valley volume (whole LRTV/GTV minus vortex volume) received a mean dose of 8 Gy, clearly under the boundaries of standard conventional palliative radiotherapy [7].

Finally, similar considerations would be considered regarding the peripheral tumor volume, where we selected the PTV volume as the dose received by the normal tissue surrounding the tumor (tissue out of the LRTV = GTV). We achieved out of the LRTV/GTV a mean dose of 5.10 Gy (within the considered PTV), which is in the upper limit of the recommended dose [19]. No additional staff was needed for the development of the plans. The mean monitor units planned in the 22 plans was 12,327 +/− 5035 (median 12,327 range 4197–22,349). Treatment time will depend on the LINAC Dose Rate (standard LINAC platform maximum 660 MU or Free Flattening Filter > 1200 MU).

Our study is limited by the reduced number of cases and the variability in tumor location treated, which threatens the generalizability of the findings. These limitations deserve future research regarding these different tumor situations. There is a strong necessity of follow-up studies to assess clinical outcomes and support future clinical applications. Furthermore, clinical outcomes analysis and comparison with standard conventional radiotherapy would improve the clinical application of this treatment. Defining additional biological immune correlative endpoints would also shed light on the clinical use of this protocol. The increased radiation time to administer an LRT session would be a limitation in some radiation oncology departments. On the other hand, the standardization of a LRT procedure [32], for debulking LRT in future combination with other cancer therapies in a wide variety of clinical situations would be relevant for future clinical practice. First, reducing tumor volume would render initially large very advanced cancers operable [33]. High doses of radiotherapy would change the tumor microenvironment, improving response to immunotherapy [6].

## 5. Conclusions

We consider that our dense LRT schedule can increase the high dose points within the tumor volume without risking the dose heterogenicity within the tumor volume, as well as respect the low doses to the tissues surrounding the tumor. This dense LRT schedule also accomplished the recommended guidelines for LRT in terms of vortex-to-valley dose ratio and OARs safe doses. Further analysis of feasibility and safety are needed to secure the clinical relevance of our proposed protocol.

## Figures and Tables

**Table 1:** Patient’s and treatment characteristics.

#	Primary TG263 Name	Treated Site	Stage	Diagnosis	LRTV (cc)	Vortex Vol (cc)	Valley Vol (cc)	Vortex Vol/LRTV	Vortex Dose (Gy)	Valley Dose (Gy)	VPDR %	Peripheral Dose (Gy)
1	Uterus Adenocarcinoma	LN_Neck_III	IV	Distant Relapse	45.35	1.75	38.89	3.86	12.14	8.69	71.52	4.17
2	Cervix SCC	Locally advanced	IIIB	Primary	114.50	8.07	104.70	7.05	11.78	7.77	65.93	4.20
3	Limb Liposarcoma	Leg_L Sarcoma	IV	Local relapse	1257.50	80.60	1178.40	6.41	11.88	8.19	68.96	5.16
4	Urachus Carcinoma	Peritoneal Carcinomatosis	IV	Local relapse	218.70	9.90	208.20	4.53	12.04	7.76	64.41	5.26
5	Tongue SCC	LN_Neck_II-III-IV	IV	Nodal relapse	127.39	4.40	121.70	3.45	12.08	7.61	63.04	4.18
6	Colon Adenocarcinoma	Peritoneal carcinomatosis	IV	Distant relapse	513.36	20.60	488.70	4.01	11.96	7.22	60.34	4.67
7	Lung Adenocarcinoma	LN_Mediastinum	IV	Nodal relapse	64.19	6.10	57.00	9.50	11.96	9.20	76.92	6.07
8	Cavity_Oral SCC	Cavity_oral	IV	Nodal relapse	335.66	22.71	307.90	6.77	11.90	8.39	70.50	6.12
9	Rectum Adenocarcinoma	Local relapse	IV	Local relapse	411.62	34.89	374.00	8.47	11.88	8.68	73.09	5.28
10	Gallbladder Adenocarcinoma	Peritoneal carcinomatosis	IV	Distant relapse	357.03	37.23	315.40	10.43	11.86	8.86	74.71	6.19
11	Sacrum Synovial Sarcoma	Local relapse	IV	Local relapse	1863.40	181.91	1674.30	9.76	11.93	9.51	79.75	6.79
12	Lung SCC	Lung left	IV	Primary	383.00	24.07	358.64	6.29	11.88	8.11	68.23	4.63
13	Colon Adenocarcinoma	Peritoneal carcinomatosis	IV	Distant Relapse	179.40	15.19	162.60	8.47	11.96	8.19	68.51	5.35
14	Ovary Epithelial Carcinoma	Peritoneal carcinomatosis	IV	Distant Relapse	377.20	22.89	351.20	6.07	11.80	7.35	62.29	5.01
15	Colon Adenocarcinoma	Peritoneal carcinomatosis	IV	Distant Relapse	316.43	28.14	285.20	8.89	11.75	9.31	79.29	5.80
16	Colon Adenocarcinoma	Peritoneal carcinomatosis	IV	Distant Relapse	156.86	14.39	140.35	9.17	11.95	8.44	70.61	4.66
17	Colon Adenocarcinoma	Peritoneal carcinomatosis	IV	Distant Relapse	219.90	21.31	196.20	9.69	11.91	8.52	71.52	5.20
18	Colon Adenocarcinoma	Liver	IV	Distant Relapse	201.47	11.83	189.50	5.87	12.05	7.43	61.64	3.16
19	Lung SCC	Lung_	IV	Primary	123.50	11.08	111.30	8.97	11.93	8.09	67.70	4.60
20	LN_Neck_II-III-IV_V Lymphoma	Lymphoma LN_Neck_II-III-IV_V	IIIB	Nodal relapse	505.20	34.62	469.30	6.85	11.96	7.05	58.99	4.22
21	LN_Neck_II-III-IV_V Nueroendocrine Carcinoma	Neuroendocrine carcinoma LN_Neck_II-III-IV_V	IV	Nodal relapse	337.50	27.51	306.30	8.15	11.96	8.89	74.37	5.42
22	Kidney_L Clear Cell Carcinoma	Local relapse	IV	Local relapse	1020.90	99.00	917.90	9.69	11.98	9.05	75.53	6.25
		**Mean** ± **SD**			415.00 ± 435.00	32.60 ± 40.70	380.00 ± 396.00	7.38 ± 2.13	11.90± 0.10	8.29 ± 0.71	5.11 ± 0.87	69.40 ± 6.02
		**Minimum–Maximum**			45.40–1863.00	1.75–1.82	38.90–1674.00	3.45–10.40	11.70–12.10	7.05–9.51	3.16–6.78	59.00–79.80
		**Median**			326.00	22.00	296.00	7.60	11.90	8.29	5.18	69.70

## Data Availability

The data presented in this study are available on request from the corresponding author.

## Supplementary Materials

The following supporting information can be downloaded at: https://www.mdpi.com/article/10.3390/cancers17122048/s1, Supplementary File S1. DVH organs at risk. Supplementary File S2. MATLAB script.
