# Supplementary material for: Automated Clinical Dosimetry Planning of Dense Lattice Radiation Therapy
Source: Cancers (Basel). 2025 Jun 19;17(12):2048. doi: 10.3390/cancers17122048 (PMC12190776; doi:10.3390/cancers17122048)
Supplement: Supplementary file 1 [file cancers-17-02048-s001.zip › cancers-3644680-supplementary.pdf]

Suppl 1. DVH ORGANS AT RISK

Case #1

Organ at risk	Volume	Min dose	Max dose	Mean dose	Modal dose	Median dose	Standard Dev
Brain	1137.7 cm^3	0.0 Gy	0.2 Gy	0.0 Gy	0.0 Gy	0.0 Gy	0.0 Gy
Mandible	69.5 cm^3	0.0 Gy	7.6 Gy	0.7 Gy	0.0 Gy	0.1 Gy	1.5 Gy
Parotid left	15.4 cm^3	0.1 Gy	7.3 Gy	1.0 Gy	0.1 Gy	0.2 Gy	1.5 Gy
Spinal cord	19.0 cm^3	0.0 Gy	4.2 Gy	0.9 Gy	0.0 Gy	0.3 Gy	1.1 Gy
Thyroid	22.6 cm^3	0.0 Gy	0.1 Gy	0.0 Gy	0.0 Gy	0.0 Gy	0.0 Gy

Case #2

Organ at risk	Volume	Min dose	Max dose	Mean dose	Modal dose	Median dose	Standard Dev
Bladder without tumor	74.9 cm^3	0.4 Gy	17.0 Gy	6.4 Gy	0.6 Gy	6.0 Gy	3.7 Gy
Bladder	86.7 cm^3	0.4 Gy	24.0 Gy	7.3 Gy	0.6 Gy	6.8 Gy	4.4 Gy
Femoral head left	127.5 cm^3	0.2 Gy	14.2 Gy	4.6 Gy	0.3 Gy	2.9 Gy	4.0 Gy
Femoral head right	133.2 cm^3	0.1 Gy	14.1 Gy	2.7 Gy	1.1 Gy	1.3 Gy	3.1 Gy
Rectum without tumor	108.9 cm^3	0.4 Gy	19.2 Gy	5.5 Gy	0.5 Gy	5.3 Gy	4.4 Gy
Rectum	122.3 cm^3	0.4 Gy	21.6 Gy	6.2 Gy	0.5 Gy	6.1 Gy	4.7 Gy

Case #3

Organ at risk	Volume	Min dose	Max dose	Mean dose	Modal dose	Median dose	Standard Dev
Bladder	138.6 cm^3	0.0 Gy	0.1 Gy	0.0 Gy	0.0 Gy	0.0 Gy	0.0 Gy
Femoral head left	167.6 cm^3	0.0 Gy	0.3 Gy	0.1 Gy	0.0 Gy	0.1 Gy	0.0 Gy
Femoral head right	179.7 cm^3	0.0 Gy	0.2 Gy	0.1 Gy	0.1 Gy	0.1 Gy	0.0 Gy
Genitalia	281.9 cm^3	0.1 Gy	9.5 Gy	1.2 Gy	0.3 Gy	0.5 Gy	1.6 Gy
Rectum	64.7 cm^3	0.0 Gy	0.3 Gy	0.1 Gy	0.0 Gy	0.1 Gy	0.1 Gy

Case #4

Organ at risk	Volume	Min dose	Max dose	Mean dose	Modal dose	Median dose	Standard Dev
Bladder	91.8 cm^3	0.4 Gy	17.3 Gy	5.0 Gy	0.9 Gy	3.6 Gy	4.0 Gy
Femoral head right	115.5 cm^3	0.1 Gy	2.9 Gy	0.3 Gy	0.1 Gy	0.2 Gy	0.4 Gy
Femoral head left	119.7 cm^3	0.1 Gy	4.4 Gy	0.6 Gy	0.1 Gy	0.2 Gy	0.8 Gy
Rectum	55.4 cm^3	0.2 Gy	10.1 Gy	2.6 Gy	0.5 Gy	1.7 Gy	2.3 Gy

Case #5

Organ at risk	Volume	Min dose	Max dose	Mean dose	Modal dose	Median dose	Standard Dev
Eye right	8.0 cm^3	0.0 Gy	0.1 Gy	0.0 Gy	0.0 Gy	0.0 Gy	0.0 Gy
Eye left	8.8 cm^3	0.0 Gy	0.0 Gy	0.0 Gy	0.0 Gy	0.0 Gy	0.0 Gy
Esophagus	5.3 cm^3	0.1 Gy	0.7 Gy	0.2 Gy	0.1 Gy	0.2 Gy	0.1 Gy
Oral cavity	33.5 cm^3	0.1 Gy	6.9 Gy	0.5 Gy	0.2 Gy	0.3 Gy	0.5 Gy
Parotid right	11.0 cm^3	0.2 Gy	23.8 Gy	2.6 Gy	0.3 Gy	0.5 Gy	4.7 Gy
PRV spinal cord	42.1 cm^3	0.1 Gy	14.2 Gy	3.0 Gy	0.1 Gy	2.8 Gy	2.6 Gy
Spinal cord	17.0 cm^3	0.1 Gy	10.1 Gy	3.0 Gy	0.1 Gy	3.3 Gy	2.5 Gy
Mandible	67.4 cm^3	0.1 Gy	15.2 Gy	2.1 Gy	0.1 Gy	0.2 Gy	3.6 Gy
Thyroid	7.7 cm^3	0.2 Gy	11.8 Gy	2.0 Gy	0.3 Gy	0.6 Gy	2.9 Gy
Trachea	12.5 cm^3	0.1 Gy	1.6 Gy	0.3 Gy	0.1 Gy	0.2 Gy	0.2 Gy

Case #6

Organ at risk	Volume	Min dose	Max dose	Mean dose	Modal dose	Median dose	Standard Dev
Bowel	1331.7 cm^3	0.5 Gy	23.7 Gy	4.8 Gy	3.7 Gy	4.4 Gy	2.5 Gy

Case #7

Organ at risk	Volume	Min dose	Max dose	Mean dose	Modal dose	Median dose	Standard Dev
Bronchial tree	11.4 cm^3	2.3 Gy	20.9 Gy	10.9 Gy	11.5 Gy	10.8 Gy	3.0 Gy
Esophagus	5.6 cm^3	2.6 Gy	11.7 Gy	7.1 Gy	5.6 Gy	7.0 Gy	1.6 Gy
Lung left	1129.1 cm^3	0.0 Gy	8.9 Gy	0.9 Gy	0.1 Gy	0.2 Gy	1.2 Gy
Lung right	1197.8 cm^3	0.1 Gy	18.7 Gy	1.8 Gy	0.1 Gy	0.4 Gy	2.3 Gy
PRV spinal cord	71.2 cm^3	0.2 Gy	10.5 Gy	3.2 Gy	0.2 Gy	3.5 Gy	2.2 Gy
Spinal cord	15.7 cm^3	0.2 Gy	7.4 Gy	3.2 Gy	0.3 Gy	3.4 Gy	2.0 Gy
Trachea	16.5 cm^3	2.5 Gy	18.8 Gy	7.2 Gy	5.5 Gy	6.6 Gy	2.7 Gy
Thyroid	21.4 cm^3	0.3 Gy	22.5 Gy	5.4 Gy	0.3 Gy	4.5 Gy	4.2 Gy

Case #8

Organ at risk	Volume	Min dose	Max dose	Mean dose	Modal dose	Median dose	Standard Dev
Mandible	89.6 cm^3	1.5 Gy	22.4 Gy	10.2 Gy	7.7 Gy	9.8 Gy	3.8 Gy
PRV spinal cord	99.5 cm^3	0.2 Gy	17.6 Gy	5.2 Gy	0.3 Gy	5.5 Gy	3.0 Gy

Spinal cord	28.8 cm^3	0.3 Gy	13.0 Gy	5.3 Gy	0.3 Gy	5.7 Gy	2.6 Gy

Case #9

Organ at risk	Volume	Min dose	Max dose	Mean dose	Modal dose	Median dose	Standard Dev
Bladder	183.7 cm^3	0.4 Gy	17.0 Gy	5.6 Gy	0.6 Gy	5.6 Gy	3.8 Gy
Femoral head left	47.3 cm^3	2.5 Gy	11.7 Gy	6.0 Gy	5.5 Gy	5.8 Gy	1.4 Gy
Femoral head right	48.3 cm^3	1.5 Gy	9.7 Gy	4.2 Gy	3.3 Gy	4.0 Gy	1.3 Gy

Case #10

Organ at risk	Volume	Min dose	Max dose	Mean dose	Modal dose	Median dose	Standard Dev
Bowel	3606.2 cm^3	0.0 Gy	21.2 Gy	2.6 Gy	0.1 Gy	1.2 Gy	3.1 Gy
Carina	2.8 cm^3	0.0 Gy	0.0 Gy	0.0 Gy	0.0 Gy	0.0 Gy	0.0 Gy
Esophagus	27.5 cm^3	0.0 Gy	0.0 Gy	0.0 Gy	0.0 Gy	0.0 Gy	0.0 Gy
Heart	744.8 cm^3	0.0 Gy	0.0 Gy	0.0 Gy	0.0 Gy	0.0 Gy	0.0 Gy
Main bronchus	4.5 cm^3	0.0 Gy	0.0 Gy	0.0 Gy	0.0 Gy	0.0 Gy	0.0 Gy
Lung left	1090.8 cm^3	0.0 Gy	0.0 Gy	0.0 Gy	0.0 Gy	0.0 Gy	0.0 Gy
Lung right	1780.8 cm^3	0.0 Gy	0.0 Gy	0.0 Gy	0.0 Gy	0.0 Gy	0.0 Gy
Lungs	2871.6 cm^3	0.0 Gy	0.0 Gy	0.0 Gy	0.0 Gy	0.0 Gy	0.0 Gy
PRV spinal cord	185.3 cm^3	0.0 Gy	4.3 Gy	0.5 Gy	0.0 Gy	0.1 Gy	0.9 Gy
SpinalCord	77.0 cm^3	0.0 Gy	4.1 Gy	0.5 Gy	0.0 Gy	0.1 Gy	0.8 Gy
Trachea	12.5 cm^3	0.0 Gy	0.0 Gy	0.0 Gy	0.0 Gy	0.0 Gy	0.0 Gy

Case #11

Organ at risk	Volume	Min dose	Max dose	Mean dose	Modal dose	Median dose	Standard Dev
Femoral head left	127.8 cm^3	0.4 Gy	21.0 Gy	5.8 Gy	0.5 Gy	4.4 Gy	4.9 Gy
Femoral head right	130.1 cm^3	0.4 Gy	25.9 Gy	6.7 Gy	0.5 Gy	3.1 Gy	6.5 Gy

Case #12

Organ at risk	Volume	Min dose	Max dose	Mean dose	Modal dose	Median dose	Standard Dev
Esophagus	16.5 cm^3	0.3 Gy	10.6 Gy	3.4 Gy	0.6 Gy	3.1 Gy	2.6 Gy
Heart	924.5 cm^3	0.2 Gy	21.2 Gy	2.2 Gy	0.3 Gy	0.8 Gy	2.6 Gy
Lung left	647.9 cm^3	0.3 Gy	19.4 Gy	4.5 Gy	0.6 Gy	4.0 Gy	3.6 Gy
Lung right	1574.9 cm^3	0.1 Gy	6.1 Gy	1.4 Gy	0.2 Gy	1.2 Gy	1.1 Gy
Lungs	2222.7 cm^3	0.1 Gy	19.4 Gy	2.3 Gy	0.2 Gy	1.5 Gy	2.6 Gy
PRV spinal cord	277.8 cm^3	0.0 Gy	3.8 Gy	0.4 Gy	0.0 Gy	0.2 Gy	0.5 Gy
Spinal cord	108.7 cm^3	0.0 Gy	3.1 Gy	0.4 Gy	0.0 Gy	0.2 Gy	0.5 Gy

Case #13

Organ at risk	Volume	Min dose	Max dose	Mean dose	Modal dose	Median dose	Standard Dev
Bladder	113.4 cm^3	0.5 Gy	19.9 Gy	4.1 Gy	0.6 Gy	3.0 Gy	3.6 Gy
Femoral head right	194.3 cm^3	0.0 Gy	13.3 Gy	0.6 Gy	0.1 Gy	0.2 Gy	1.4 Gy
Femoral head left	212.7 cm^3	0.0 Gy	11.8 Gy	0.5 Gy	0.0 Gy	0.2 Gy	1.1 Gy
Rectum	38.7 cm^3	0.1 Gy	3.3 Gy	0.5 Gy	0.2 Gy	0.4 Gy	0.4 Gy

Case #14

Organ at risk	Volume	Min dose	Max dose	Mean dose	Modal dose	Median dose	Standard Dev
Bladder	26.2 cm^3	0.9 Gy	19.9 Gy	3.3 Gy	1.2 Gy	1.6 Gy	3.7 Gy
Femoral head right	165.3 cm^3	0.1 Gy	12.3 Gy	0.6 Gy	0.1 Gy	0.2 Gy	1.3 Gy
Femoral head left	173.8 cm^3	0.0 Gy	13.1 Gy	0.8 Gy	0.1 Gy	0.2 Gy	1.9 Gy
Rectum	53.4 cm^3	0.2 Gy	18.7 Gy	4.3 Gy	0.3 Gy	1.3 Gy	5.0 Gy

Case #15

Organ at risk	Volume	Min dose	Max dose	Mean dose	Modal dose	Median dose	Standard Dev
Kidney right	119.0 cm^3	1.1 Gy	19.5 Gy	5.9 Gy	4.1 Gy	5.3 Gy	2.7 Gy
Kidney left	66.5 cm^3	0.8 Gy	14.6 Gy	3.8 Gy	3.1 Gy	3.5 Gy	1.6 Gy
Liver	146.1 cm^3	0.8 Gy	24.9 Gy	7.0 Gy	4.4 Gy	6.1 Gy	4.3 Gy
PRV spinal cord	97.4 cm^3	0.3 Gy	12.8 Gy	4.7 Gy	0.4 Gy	4.8 Gy	3.1 Gy
SpinalCord	36.1 cm^3	0.3 Gy	12.0 Gy	4.8 Gy	0.4 Gy	4.8 Gy	3.1 Gy

Case #16

Organ at risk	Volume	Min dose	Max dose	Mean dose	Modal dose	Median dose	Standard Dev
Kidney left	112.9 cm^3	0.2 Gy	3.0 Gy	1.0 Gy	0.3 Gy	1.1 Gy	0.5 Gy
Kidney right	116.4 cm^3	0.2 Gy	6.6 Gy	1.5 Gy	0.3 Gy	1.4 Gy	0.9 Gy
Kidneys	229.3 cm^3	0.2 Gy	6.6 Gy	1.2 Gy	0.3 Gy	1.2 Gy	0.8 Gy
Liver	76.2 cm^3	0.2 Gy	18.4 Gy	1.0 Gy	0.3 Gy	0.4 Gy	2,1 Gy

Case #17

Organ at risk	Volume	Min dose	Max dose	Mean dose	Modal dose	Median dose	Standard Dev
Kidney left	116.5 cm^3	0.0 Gy	0.1 Gy	0.0 Gy	0.0 Gy	0.0 Gy	0.0 Gy
Kidney right	131.9 cm^3	0.0 Gy	0.1 Gy	0.0 Gy	0.0 Gy	0.0 Gy	0.0 Gy
Liver	974.1 cm^3	0.0 Gy	0.0 Gy	0.0 Gy	0.0 Gy	0.0 Gy	0.0 Gy
PRV spinal cord	109.9 cm^3	0.0 Gy	0.4 Gy	0.1 Gy	0.0 Gy	0.0 Gy	0.1 Gy
Stomach	262.8 cm^3	0.0 Gy	0.1 Gy	0.0 Gy	0.0 Gy	0.0 Gy	0.0 Gy
Spinal cord	33.4 cm^3	0.0 Gy	0.3 Gy	0.0 Gy	0.0 Gy	0.0 Gy	0.1 Gy

Case #18

Organ at risk	Volume	Min dose	Max dose	Mean dose	Modal dose	Median dose	Standard Dev
Kidney right	128.1 cm^3	0.1 Gy	7.7 Gy	0.5 Gy	0.2 Gy	0.4 Gy	0.4 Gy
Kidney left	176.4 cm^3	0.1 Gy	5.3 Gy	1.4 Gy	0.1 Gy	0.4 Gy	1.5 Gy
Kidneys	304.5 cm^3	0.1 Gy	7.7 Gy	1.0 Gy	0.1 Gy	0.4 Gy	1.2 Gy
Liver	930.1 cm^3	0.1 Gy	26.1 Gy	3.3 Gy	0.3 Gy	0.8 Gy	4.4 Gy
Lung left	1648.1 cm^3	0.0 Gy	1.1 Gy	0.1 Gy	0.0 Gy	0.1 Gy	0.1 Gy
Lung right	1912.1 cm^3	0.0 Gy	6.9 Gy	0.1 Gy	0.0 Gy	0.1 Gy	0.1 Gy
Lungs	3560.2 cm^3	0.0 Gy	6.9 Gy	0.1 Gy	0.0 Gy	0.1 Gy	0.1 Gy
PRV Spinal cord	93.6 cm^3	0.2 Gy	14.4 Gy	2.9 Gy	0.2 Gy	1.0 Gy	3.0 Gy
Spinal cord	36.4 cm^3	0.2 Gy	13.6 Gy	3.2 Gy	0.2 Gy	1.7 Gy	3.1 Gy

Case #19

Organ at risk	Volume	Min dose	Max dose	Mean dose	Modal dose	Median dose	Standard Dev
Heart	640.3 cm^3	0.1 Gy	25.2 Gy	3.1 Gy	0.2 Gy	0.4 Gy	5.8 Gy
Lung left	2462.0 cm^3	0.0 Gy	30.4 Gy	1.5 Gy	0.0 Gy	0.3 Gy	3.5 Gy
Lung right	2711.1 cm^3	0.0 Gy	11.6 Gy	0.8 Gy	0.0 Gy	0.1 Gy	1.7 Gy
Lungs	5173.1 cm^3	0.0 Gy	30.4 Gy	1.1 Gy	0.0 Gy	0.2 Gy	2.8 Gy
PRV spinal cord	74.1 cm^3	0.1 Gy	13.0 Gy	1.0 Gy	0.2 Gy	0.5 Gy	1.6 Gy
Spinal cord	35.5 cm^3	0.2 Gy	11.5 Gy	0.9 Gy	0.2 Gy	0.5 Gy	1.0 Gy

Case #20

Organ at risk	Volume	Min dose	Max dose	Mean dose	Modal dose	Median dose	Standard Dev
Cerebrum	1220.8 cm^3	0.0 Gy	11.2 Gy	0.4 Gy	0.1 Gy	0.1 Gy	0.9 Gy
Eye left	7.8 cm^3	0.1 Gy	0.3 Gy	0.2 Gy	0.1 Gy	0.2 Gy	0.0 Gy
Eye right	9.5 cm^3	0.1 Gy	0.2 Gy	0.2 Gy	0.1 Gy	0.2 Gy	0.0 Gy
Larynx	60.7 cm^3	2.5 Gy	28.3 Gy	7.5 Gy	5.5 Gy	6.6 Gy	3.7 Gy
Madible	90.9 cm^3	0.4 Gy	22.7 Gy	3.9 Gy	1.8 Gy	2.9 Gy	2.9 Gy
PRV Spinal cord	77.9 cm^3	0.3 Gy	18.6 Gy	5.5 Gy	0.4 Gy	5.1 Gy	3.7 Gy
Spinal cord	37.6 cm^3	0.3 Gy	17.9 Gy	5.4 Gy	0.4 Gy	5.1 Gy	3.6 Gy
Thyroid	9.9 cm^3	2.4 Gy	23.6 Gy	8.3 Gy	9.6 Gy	8.1 Gy	3.7 Gy

Case #21

Organ at risk	Volume	Min dose	Max dose	Mean dose	Modal dose	Median dose	Standard Dev
Esophagus	4.4 cm^3	0.7 Gy	12.7 Gy	4.5 Gy	0.8 Gy	3.8 Gy	3.4 Gy
Eye right	8.4 cm^3	0.0 Gy	0.1 Gy	0.1 Gy	0.1 Gy	0.1 Gy	0.0 Gy
Eye left	8.9 cm^3	0.0 Gy	0.1 Gy	0.1 Gy	0.1 Gy	0.1 Gy	0.0 Gy
Thyroid	10.5 cm^3	4.4 Gy	32.8 Gy	16.9 Gy	17.4 Gy	17.0 Gy	5.6 Gy
Trachea	17.0 cm^3	0.6 Gy	17.4 Gy	3.7 Gy	0.6 Gy	3.4 Gy	2.6 Gy
Larynx	37.0 cm^3	3.7 Gy	26.4 Gy	9.7 Gy	4.0 Gy	8.6 Gy	4.4 Gy
Spinal cord	38.9 cm^3	0.4 Gy	17.8 Gy	5.4 Gy	0.7 Gy	5.3 Gy	3.5 Gy
PRV spinal cord	81.4 cm^3	0.4 Gy	18.9 Gy	5.3 Gy	0.6 Gy	4.9 Gy	3.6 Gy
Lung right	2085.3 cm^3	0.0 Gy	6.0 Gy	0.2 Gy	0.0 Gy	0.1 Gy	0.3 Gy
Lung left	1390.7 cm^3	0.0 Gy	20.2 Gy	0.5 Gy	0.0 Gy	0.3 Gy	1.1 Gy
Cerebrum	1421.6 cm^3	0.0 Gy	0.6 Gy	0.1 Gy	0.0 Gy	0.1 Gy	0.1 Gy

Case #22

Organ at risk	Volume	Min dose	Max dose	Mean dose	Modal dose	Median dose	Standard Dev
Spinal cord	38.0 cm^3	0.6 Gy	12.6 Gy	4.8 Gy	1.1 Gy	4.9 Gy	2.9 Gy
PRV spinal cord	83.3 cm^3	0.5 Gy	14.4 Gy	4.6 Gy	1.0 Gy	4.6 Gy	3.1 Gy
Heart	661.8 cm^3	0.0 Gy	0.3 Gy	0.1 Gy	0.0 Gy	0.1 Gy	0.1 Gy
Bowel	1860.4 cm^3	0.6 Gy	25.2 Gy	8.1 Gy	1.9 Gy	7.0 Gy	5.6 Gy
Lung left	1876.2 cm^3	0.0 Gy	0.6 Gy	0.1 Gy	0.0 Gy	0.0 Gy	0.1 Gy

Lung right	2627.1 cm^3	0.0 Gy	1.0 Gy	0.1 Gy	0.0 Gy	0.0 Gy	0.2 Gy
Lungs	4503.3 cm^3	0.0 Gy	1.0 Gy	0.1 Gy	0.0 Gy	0.0 Gy	0.1 Gy
Liver	2198.6 cm^3	0.0 Gy	35.0 Gy	1.5 Gy	0.0 Gy	0.6 Gy	3.3 Gy

Suppl 2.- MATLAB SCRIPT

```
%%%%%%%%%%%%%%%%%%%%%%%%%%%%%%%%%%%%%%%%%%%%%%%%%%%%%%%%%%%%%%%%%%%%%%%%%%%%%%%%%%%%%%%%%%%%%%%%%%%%%%
%%%%%%%%%%%%%%%%%%%%%%%%%%%%%%%%%%%%%%%%%%%%%%%%%%%%%%%%%%%%%%%%%%%%%%%%%%%%%%%%%%%%%%%%%%%%%%%%%%%%%%
%%%%%%%%%%%%%%%%%%%%%%%%%%%%%%%%%%%%%%%%%%%%%%%%%%%%%%%%%%%%%%%%%%%%%%%%%%%%%%%%%%%%%%%%%%%%%%%%%%%%%%

% This script reads a dose image in dicom format exported from Eclipse TPS (Varian),
% plots it in both 3D and gradient image, and draws X, Y and diagonal profiles at the same time,
% estimating the distances between maxima and minima.

%%%%%%%%%%%%%%%%%%%%%%%%%%%%%%%%%%%%%%%%%%%%%%%%%%%%%%%%%%%%%%%%%%%%%%%%%%%%%%%%%%%%%%%%%%%%%%%%%%%%%%
%%%%%%%%%%%%%%%%%%%%%%%%%%%%%%%%%%%%%%%%%%%%%%%%%%%%%%%%%%%%%%%%%%%%%%%%%%%%%%%%%%%%%%%%%%%%%%%%%%%%%%
%%%%%%%%%%%%%%%%%%%%%%%%%%%%%%%%%%%%%%%%%%%%%%%%%%%%%%%%%%%%%%%%%%%%%%%%%%%%%%%%%%%%%%%%%%%%%%%%%%%%%%

% Read a dicom image containing

clear all;
close all;
clc;

clear

% Specify the default folder and file filter

defaultFolder = % define;

[filename, pathName] = uigetfile({'*.m;*.mat;*.fig; *.dcm', 'MATLAB or DICOM Files (*.m, *.mat,
*.fig)'; ...
                                   '*.*', 'All Files (*.*)'}, ...
                                   'Select a file to open', defaultFolder);

% Specify the folder containing the files

folderPath = pathName;

% Get a list of all .txt files in the folder

filePattern = fullfile(folderPath, '*.dcm'); % Change the extension as needed
files = dir(filePattern);
filesSet = files(:).name;

% Open a file to write at the end in an Excel file

folderResults = '/Users/davidmacias-verde/Documents/MATLAB/LatexResults/';
fileID = strcat(folderResults, 'Table-Latex.xlsx');

% Initialize a table to hold the results

data = table();

% Loop through each file and open it

%for k = 1:length(files)

for k = 1:1

% Get the full dicom file name

fileName = files(k).name

fullFileName = fullfile(folderPath, fileName);

% Read the DICOM file

imageData = dicomread(fullFileName);
z = imageData;

% Read the DICOM metadata
info = dicominfo(fullFileName);

% Extract pixel spacing

pixelSpacingF = info.PixelSpacing; % Pixel spacing in mm
pixelSpacing = pixelSpacingF(1);

% Remove outbounds

z(:, 1:3) = [];
[rows, cols] = size(z);
z(:, cols-3:cols) = [];
[cols, rows] = size(z);
```

```
% Create coordinate grids in physical units
% The x's are the columns and the y's are the rows, they are inverted to how they are represented with a plot later on
% where the x's are the abscissae and the y's are the ordinates.

[cols, rows] = size(z);
[xpu, ypu] = meshgrid((1:cols) * pixelSpacing, (1:rows) * pixelSpacing);


%%%%%%%%%%%%%%%%%%%%%%%%%%%%%%%%%%%%%%%%%%%%%%%%%%%%%%%%%%%%%%%%%%%%%%%%%%%%%%%%%%%%%%%%%%%%%%%%
%%%%%%%%%%%%%%%%%%%%%%%%%%%%%%%%%%%%%%%%%%%%%%%%%%%%%%%%%%%%%%%%%%%%%%%%%%%%%%%%%%%%%%%%%%%%%%%%
%%%%%%%%%%%%%%%%%%%%%%%%%%%%%%%%%%%%%%%%%%%%%%%%%%%%%%%%%%%%%%%%%%%%%%%%%%%%%%%%%%%%%%%%%%%%%%%%

% Show image as a surface figure

figure;

s = surf(xpu, ypu, z./10000);
axis on;
axis padded;
cb = colorbar;
colormap(jet)
zlabel('Relative dose')
xlabel('mm');
ylabel('mm');

xlim([0 rows*pixelSpacing]);
ylim([0 cols*pixelSpacing]);
yticks(0:5:rows*pixelSpacing);
xticks(0:5:cols*pixelSpacing);

azimuth = 30;
elevation = 70;
view(azimuth, elevation); % Apply the view settings
s.EdgeColor = 'none';
axis equal; % Maintain equal scaling
ax = gca;
ax.FontSize = 28;

figureName = strcat(folderResults, fileName, '-surf.pdf');
exportgraphics(gcf, figureName);
close(gcf);

%%%%%%%%%%%%%%%%%%%%%%%%%%%%%%%%%%%%%%%%%%%%%%%%%%%%%%%%%%%%%%%%%%%%%%%%%%%%%%%%%%%%%%%%%%%%%%%%
%%%%%%%%%%%%%%%%%%%%%%%%%%%%%%%%%%%%%%%%%%%%%%%%%%%%%%%%%%%%%%%%%%%%%%%%%%%%%%%%%%%%%%%%%%%%%%%%
%%%%%%%%%%%%%%%%%%%%%%%%%%%%%%%%%%%%%%%%%%%%%%%%%%%%%%%%%%%%%%%%%%%%%%%%%%%%%%%%%%%%%%%%%%%%%%%%

% Show image as a gradient figure

figure;

% Compute the gradient components

[Gx, Gy] = imgradientxy(z);

% Define the number of contour levels

numLevels = 10; % Specify the number of levels
contourf(xpu, ypu, z/10000, numLevels);
cb = colorbar;
axis on; % Turn on the axis
axis padded;
colormap(jet)
zlabel('Relative dose')
xlabel('mm');
ylabel('mm');

xlim([0 rows*pixelSpacing]);
ylim([0 cols*pixelSpacing]);
yticks(0:5:rows*pixelSpacing);
xticks(0:5:cols*pixelSpacing);

xtickangle(90);
ax = gca;
ax.FontSize = 28;
axis equal; % Maintain equal scaling
grid on;
hold on;
```

```
%%%%%%%%%%%%%%%%%%%%%%%%%%%%%%%%%%%%%%%%%%%%%%%%%%%%%%%%%%%%%%%%%%%%%%%%%%%%%%%%%%%%%%%%%%%%%%%%
%%%%%%%%%%%%%%%%%%%%%%%%%%%%%%%%%%%%%%%%%%%%%%%%%%%%%%%%%%%%%%%%%%%%%%%%%%%%%%%%%%%%%%%%%%%%%%%%
%%%%%%%%%%%%%%%%%%%%%%%%%%%%%%%%%%%%%%%%%%%%%%%%%%%%%%%%%%%%%%%%%%%%%%%%%%%%%%%%%%%%%%%%%%%%%%%%

% Kernel definitoion for local maxima and minima detection

kernel = ones(25, 25);

% Kernal convolution

convMatrix = conv2(z, kernel, 'same');

% Find local maxima and minima

localMax = islocalmax2(convMatrix);
localMin = islocalmin2(convMatrix);

% Extract max and min coordinate

[xMax, yMax] = find(localMax);
[xMin, yMin] = find(localMin);

maxCoords = [xMax, yMax]*pixelSpacing;
minCoords = [xMin, yMin]*pixelSpacing;

% Plot markers over max and min points

plot(yMax*pixelSpacing, xMax*pixelSpacing, 'ro', 'MarkerSize', 30, 'LineWidth', 2); % Local max
points
hold on;

plot(yMin*pixelSpacing, xMin*pixelSpacing, 'bo', 'MarkerSize', 30, 'LineWidth', 2); % Local min
points
hold on;

% Add value name to the max and min points

for i = 1:length(xMax)
    text(yMax(i)*pixelSpacing, xMax(i)*pixelSpacing, num2str(z(xMax(i), yMax(i))/10000), 'Color',
'white', 'FontSize', 16, 'HorizontalAlignment', 'center');
end

for i = 1:length(xMin)
    text(yMin(i)*pixelSpacing, xMin(i)*pixelSpacing, num2str(z(xMin(i), yMin(i))/10000), 'Color',
'black', 'FontSize', 16, 'HorizontalAlignment', 'center');
end

hold on;

%%%%%%%%%%%%%%%%%%%%%%%%%%%%%%%%%%%%%%%%%%%%%%%%%%%%%%%%%%%%%%%%%%%%%%%%%%%%%%%%%%%%%%%%%%%%%%%%
%%%%%%%%%%%%%%%%%%%%%%%%%%%%%%%%%%%%%%%%%%%%%%%%%%%%%%%%%%%%%%%%%%%%%%%%%%%%%%%%%%%%%%%%%%%%%%%%
%%%%%%%%%%%%%%%%%%%%%%%%%%%%%%%%%%%%%%%%%%%%%%%%%%%%%%%%%%%%%%%%%%%%%%%%%%%%%%%%%%%%%%%%%%%%%%%%

% X and Y profiles

numeroObjetivoX = round(median(maxCoords(:,1)));
numeroObjetivoY = round(median(maxCoords(:,2)));

distanciasX = abs(maxCoords(:,1) - numeroObjetivoX);
distanciasY = abs(maxCoords(:,2) - numeroObjetivoY);

% Encontrar el índice del valor más cercano

[~, indiceCercanoX] = min(distanciasX);
[~, indiceCercanoY] = min(distanciasY);

% Get nearest points

valorCercanoX = maxCoords(indiceCercanoX,1);
valorCercanoY = maxCoords(indiceCercanoY,2);

plot([1, size(z, 2)*pixelSpacing], [valorCercanoX, valorCercanoX], 'b', 'LineWidth', 2);
plot([valorCercanoY, valorCercanoY], [1, size(z, 1)*pixelSpacing], 'b', 'LineWidth', 2);

%%%%%%%%%%%%%%%%%%%%%%%%%%%%%%%%%%%%%%%%%%%%%%%%%%%%%%%%%%%%%%%%%%%%%%%%%%%%%%%%%%%%%%%%%%%%%%%%
%%%%%%%%%%%%%%%%%%%%%%%%%%%%%%%%%%%%%%%%%%%%%%%%%%%%%%%%%%%%%%%%%%%%%%%%%%%%%%%%%%%%%%%%%%%%%%%%
%%%%%%%%%%%%%%%%%%%%%%%%%%%%%%%%%%%%%%%%%%%%%%%%%%%%%%%%%%%%%%%%%%%%%%%%%%%%%%%%%%%%%%%%%%%%%%%%
```

```
% Diagonal profile

M = maxCoords(:,1)+maxCoords(:,2);

[maxValue, maxIndex] = max(M);
[minValue, minIndex] = min(M);

% Get two points defining diagonal line

Start = maxCoords(minIndex,:);
End = maxCoords(maxIndex,:);

diagonalPoints = [Start; End];

x1 = Start(1); y1 = Start(2);
x2 = End(1); y2 = End(2);

% Define a set of points

points = maxCoords;

% Calculate the coefficients of the line equation (Ax + By + C = 0)

A = y2 - y1; % slope component y
B = x1 - x2; % slope component x
C = x2*y1 - x1*y2; % constant term

% Specify the minimum distance threshold

minDistanceThreshold = 5;

% Initialize an array to store distances

distances = zeros(size(points, 1), 1);

% Loop through each point to calculate distances

for i = 1:size(points, 1)

    x0 = points(i, 1);
    y0 = points(i, 2);

    % Calculate the distance from the point to the line

    distances(i) = abs(A*x0 + B*y0 + C) / sqrt(A^2 + B^2);
end

% Filter points based on the minimum distance threshold

filteredPoints = points(distances <= minDistanceThreshold, :);

rowsDiag = size(filteredPoints,1);
colsDiag = size(filteredPoints,2);

num_diagonal_points = min(rowsDiag, colsDiag);

diagonal_points = zeros(num_diagonal_points, 2); % Preallocate for efficiency

for i = 1:num_diagonal_points
    diagonal_points(i, :) = [i, i]; % (row, column) coordinates
end

% Extract x and y coordinates

x = filteredPoints(:, 2);
y = filteredPoints(:, 1);

% Plot the line connecting the points

plot(x, y, 'b', 'LineWidth', 2);
ax = gca;
ax.FontSize = 28;

figureName = strcat(folderResults, fileName, '-contour.pdf');
exportgraphics(gcf, figureName);
close(gcf);

%%%%%%%%%%%%%%%%%%%%%%%%%%%%%%%%%%%%%%%%%%%%%%%%%%%%%%%%%%%%%%%%%%%%%%%%%%%%%%%%%%%%%%%%%%%%%%%%%
%%%%%%%%%%%%%%%%%%%%%%%%%%%%%%%%%%%%%%%%%%%%%%%%%%%%%%%%%%%%%%%%%%%%%%%%%%%%%%%%%%%%%%%%%%%%%%%%
```

```
%%%%%%%%%%%%%%%%%%%%%%%%%%%%%%%%%%%%%%%%%%%%%%%%%%%%%%%%%%%%%%%%%%%%%%%%%%%%%%%%%%%%%%%%%%%%%%%%%

mPHeight = 95;  % Default value
mPDistance = 10;  % Default value
mPProminence = 2;  % Default value
wsize = 25;  % Default value

%%%%%%%%%%%%%%%%%%%%%%%%%%%%%%%%%%%%%%%%%%%%%%%%%%%%%%%%%%%%%%%%%%%%%%%%%%%%%%%%%%%%%%%%%%%%%%%%%
%%%%%%%%%%%%%%%%%%%%%%%%%%%%%%%%%%%%%%%%%%%%%%%%%%%%%%%%%%%%%%%%%%%%%%%%%%%%%%%%%%%%%%%%%%%%%%%%%
%%%%%%%%%%%%%%%%%%%%%%%%%%%%%%%%%%%%%%%%%%%%%%%%%%%%%%%%%%%%%%%%%%%%%%%%%%%%%%%%%%%%%%%%%%%%%%%%%

% Profile line in X constant

startH = 1;
endH = cols;
pixelValuesX = z(valorCercanoX/pixelSpacing,startH:endH)./10000;

figure;

coordX = startH*pixelSpacing:pixelSpacing:endH*pixelSpacing;
[Xx,Yx] = meshgrid(valorCercanoX/pixelSpacing, coordX/pixelSpacing);
pixelValuesX = improfile(double(z)/10000, Yx, Xx);
plot(coordX, pixelValuesX, 'b*');
ax = gca;
ax.FontSize = 28;
hold on;

% Smooth the signal using a moving average. Size of the moving average window

windowSize = wsize; % Size of the moving average window
smoothedSignalX = movmean(pixelValuesX, windowSize);

% Create a smoothing spline fit

splineFit = fit(coordX', smoothedSignalX, 'smoothingspline');

% Generate finer x values for evaluation

steps = 5000;
xi = coordX;
yi = feval(splineFit, coordX);
plot(xi, yi)

xlabel('Image side size mm');
ylabel('Relative Dose (%)');
xtickangle(90);
legend('Data Points', 'Smoothing Spline','FontSize', 28,
'Location','southoutside','Orientation','horizontal');
title('Patient axial profile over axial dose plane')
ax = gca;
ax.FontSize = 28;
grid on;
hold off;

% Find peaks and valleys in the spline

[peaksX, peakLocationsX] = findpeaks(yi, 'MinPeakHeight', mPHeight,'MinPeakDistance',
mPDistance/pixelSpacing, 'MinPeakProminence', mPProminence);
[valleysX, valleyLocationsX] = findpeaks(-yi, 'MinPeakDistance', mPDistance/pixelSpacing,
'MinPeakProminence', mPProminence);

hold on;

plot(xi(peakLocationsX), peaksX, 'bo', 'MarkerSize', 25, 'DisplayName', 'Peaks','LineWidth',2);
plot(xi(valleyLocationsX), -valleysX, 'ro', 'MarkerSize', 25, 'DisplayName',
'Valleys','LineWidth',2);

figureName = strcat(folderResults, fileName, '-axial.pdf');
exportgraphics(gcf, figureName);
close(gcf);

% Calculate the distance between the maximum peaks

try

for i=1:length(peakLocationsX)-1
    distanceMaxX(i) = abs(xi(peakLocationsX(i+1))- xi(peakLocationsX(i)));
end
```

```
Calculate the distance between the minimum peaks

for j=1:length(valleyLocationsX)-1
    distanceMinX(j) = abs(xi(valleyLocationsX(j+1)) - xi(valleyLocationsX(j)));
end

meandistmaxX = min(mean(distanceMaxX));
meandistminX = min(mean(distanceMinX));

catch ME

    meandistmaxX = 0;
    meandistminX = 0;

    fprintf('An error occurred: %s\n', ME.message);

end

meanmaxX = mean (peaksX);
meanminX = mean (-valleysX);
VPDRX = (meanminX / meanmaxX)*100;

%%%%%%%%%%%%%%%%%%%%%%%%%%%%%%%%%%%%%%%%%%%%%%%%%%%%%%%%%%%%%%%%%%%%%%%%%%%%%%%%%%%%%%%%%%%%%%%%
%%%%%%%%%%%%%%%%%%%%%%%%%%%%%%%%%%%%%%%%%%%%%%%%%%%%%%%%%%%%%%%%%%%%%%%%%%%%%%%%%%%%%%%%%%%%%%%%
%%%%%%%%%%%%%%%%%%%%%%%%%%%%%%%%%%%%%%%%%%%%%%%%%%%%%%%%%%%%%%%%%%%%%%%%%%%%%%%%%%%%%%%%%%%%%%%%

% Profile line in Y constant

startV = 1;
endV = rows;

figure;

coordY = startV*pixelSpacing:pixelSpacing:endV*pixelSpacing;
[Xy,Yy] = meshgrid(coordY/pixelSpacing,valorCercanoY/pixelSpacing);
pixelValuesY = improfile(double(z)/10000, Yy, Xy);
plot(coordY, pixelValuesY, 'b*');
hold on;

% Smooth the signal using a moving average

windowSize = 10; % Size of the moving average window

smoothedSignalY = movmean(pixelValuesY, windowSize);

% Create a smoothing spline fit

splineFit = fit(coordY', smoothedSignalY, 'smoothingspline');

% Generate finer x values for evaluation

steps = 5000;
xi = coordY;
yi = feval(splineFit, xi);
plot(xi, yi)

ax = gca;
ax.FontSize = 28;
xtickangle(90);
legend('Data Points', 'Smoothing Spline','FontSize', 28,
'Location','southoutside','Orientation','horizontal');
title('Patient coronal profile over axial dose plane');
grid on;
hold off;

% Find peaks and valleys in the spline

[peaksY, peakLocationsY] = findpeaks(yi, 'MinPeakHeight', mPHeight,'MinPeakDistance',
mPDistance/pixelSpacing, 'MinPeakProminence', mPProminence);
[valleysY, valleyLocationsY] = findpeaks(-yi, 'MinPeakDistance', mPDistance/pixelSpacing,
'MinPeakProminence', mPProminence);

hold on;

plot(xi(peakLocationsY), peaksY, 'bo', 'MarkerSize', 25, 'DisplayName', 'Peaks','LineWidth',2);
plot(xi(valleyLocationsY), -valleysY, 'ro', 'MarkerSize', 25, 'DisplayName',
'Valleys','LineWidth',2);

figureName = strcat(folderResults, fileName, '-sagittal.pdf');
```

```
exportgraphics(gcf, figureName);
close(gcf);

% Calculate the distance between the maximum peaks

try

for i=1:length(peakLocationsY)-1
    distanceMaxY(i) = abs(xi(peakLocationsY(i+1))- xi(peakLocationsY(i)));
end

Calculate the distance between the minimum peaks

for j=1:length(valleyLocationsY)-1
    distanceMinY(j) = abs(xi(valleyLocationsY(j+1)) - xi(valleyLocationsY(j)));
end

meandistmaxY = min(mean(distanceMaxY));
meandistminY = min(mean(distanceMinY));

catch ME

    meandistmaxY = 0;
    meandistminY = 0;

    fprintf('An error occurred: %s\n', ME.message);

end

meanmaxY = mean (peaksY);
meanminY = mean (-valleysY);
VPDRY = (meanminY / meanmaxY)*100;

%%%%%%%%%%%%%%%%%%%%%%%%%%%%%%%%%%%%%%%%%%%%%%%%%%%%%%%%%%%%%%%%%%%%%%%%%%%%%%%%%%%%%%%%%%%%%%%
%%%%%%%%%%%%%%%%%%%%%%%%%%%%%%%%%%%%%%%%%%%%%%%%%%%%%%%%%%%%%%%%%%%%%%%%%%%%%%%%%%%%%%%%%%%%%%%
%%%%%%%%%%%%%%%%%%%%%%%%%%%%%%%%%%%%%%%%%%%%%%%%%%%%%%%%%%%%%%%%%%%%%%%%%%%%%%%%%%%%%%%%%%%%%%%

% Profile along diagonal line

Xd = filteredPoints(:,1);
Yd = filteredPoints(:,2);

pixelSpacingD = sqrt(2*pixelSpacing^2);

figure;

pixelValuesD = improfile(double(z)/10000, Yd./pixelSpacing, Xd./pixelSpacing);
coordD = 1:size(pixelValuesD,1);
plot(coordD*pixelSpacing, pixelValuesD, 'b*');
hold on;

% Smooth the signal using a moving average. Size of the moving average window

windowSize = wsize;
smoothedSignalD = movmean(pixelValuesD, windowSize);

steps = 5000;
x_fit = linspace(1, size(pixelValuesD,1), steps);

% Estimate the period (optional)

[acor, lag] = xcorr(smoothedSignalD - mean(smoothedSignalD), 'coeff');
[~, idx] = max(acor(lag > 0));
period_est = xi(find(lag > 0, 1) + idx - 1);

% Periodo

fprintf('Estimated period: %.3f seconds\n', period_est);

% Thresholding approach to detect steps ---

threshold = (max(smoothedSignalD) + min(smoothedSignalD)) / 2;
is_high = smoothedSignalD > threshold;

% Compute average values in high and low states ---

A_high = mean(smoothedSignalD(is_high));
A_low  = mean(smoothedSignalD(~is_high));
```

```
% Generate fitted step signal

y_fit = zeros(size(x_fit));
y_fit(is_high) = A_high;
y_fit(~is_high) = A_low;

% Plot original and fitted signal

plot(coordD*pixelValuesD, pixelValuesD, 'b', 'DisplayName', 'Original signal');

hold on;

plot(x_fit*pixelSpacingD, y_fit);

xlabel('Image side size mm');
ylabel('Relative Dose (%)');
xtickangle(90);
legend('Data Points', 'Smoothing Spline','FontSize', 28,
'Location','southoutside','Orientation','horizontal');
title('Patient diagonal (axial + coronal) profile over axial dose plane')
ax = gca;
ax.FontSize = 28;
grid on;
hold off;

% Find peaks and valleys in the spline

[peaksD, peakLocationsD] = findpeaks(y_fit, 'MinPeakHeight', mPHeight,'MinPeakDistance',
mPDistance/pixelSpacingD, 'MinPeakProminence', mPProminence);
[valleysD, valleyLocationsD] = findpeaks(-y_fit, 'MinPeakHeight', -80, 'MinPeakDistance',
mPDistance/pixelSpacingD, 'MinPeakProminence', mPProminence);

% Identify them in the plot

hold on;

plot(x_fit(peakLocationsD)*pixelSpacingD, peaksD, 'bo', 'MarkerSize', 25, 'DisplayName',
'Peaks','LineWidth',2);
plot(x_fit(valleyLocationsD)*pixelSpacingD, -valleysD, 'ro', 'MarkerSize', 25, 'DisplayName',
'Valleys','LineWidth',2);

figureName = strcat(folderResults, fileName, '-diagonal.pdf');
exportgraphics(gcf, figureName);
close(gcf);

% Calculate distance between maximum peaks

try

for i=1:length(peakLocationsD)-1
    distanceMaxD(i) = abs(x_fit(peakLocationsD(i+1))- x_fit(peakLocationsD(i)))*pixelSpacingD;
end

% Calculate distance between minimum peaks

for j=1:length(valleyLocationsD)-1
    distanceMinD(j) = abs(x_fit(valleyLocationsD(j+1)) - x_fit(valleyLocationsD(j)))*pixelSpacingD;
end

meandistmaxD = min(mean(distanceMaxD));
meandistminD = min(mean(distanceMinD));

catch ME

    meandistmaxD = 0;
    meandistminD = 0;

    fprintf('An error occurred: %s\n', ME.message);

end

meanmaxD = mean (peaksD);
meanminD = mean (-valleysD);
VPDRD = (meanminD / meanmaxD)*100;

% Create a new row of data
newRow = table(k, meandistmaxX, meandistminX, meanmaxX, meanminX, VPDRX, ...
            meandistmaxY, meandistminY, meanmaxY, meanminY, VPDRY, ...
            meandistmaxD, meandistminD, meanmaxD, meanminD, VPDRD, ...
```

```
            'VariableNames', {'Case', 'Axial max dist', 'Axial min dist', 'Axial mean max', 'Axial
mean min', 'Axial VPDR', ...
            'Sagittal max dist', 'Sagittal min dist', 'Sagittal mean max', 'Sagitall mean min',
'Sagittal VPDR', ...
            'Diagonal max dist', 'Diagonal min dist', 'Diagonal mean max', 'Diagonal mean min',
'Diagonal VPDR'});

% Append the new row to the existing table
data = [data; newRow];

end

%%%%%%%%%%%%%%%%%%%%%%%%%%%%%%%%%%%%%%%%%%%%%%%%%%%%%%%%%%%%%%%%%%%%%%%%%%%%%%%%%%%%%%%%%%%%%%%%
%%%%%%%%%%%%%%%%%%%%%%%%%%%%%%%%%%%%%%%%%%%%%%%%%%%%%%%%%%%%%%%%%%%%%%%%%%%%%%%%%%%%%%%%%%%%%%%%
%%%%%%%%%%%%%%%%%%%%%%%%%%%%%%%%%%%%%%%%%%%%%%%%%%%%%%%%%%%%%%%%%%%%%%%%%%%%%%%%%%%%%%%%%%%%%%%%

% Write the table to an Excel file

excelFileName = strcat(folderResults, 'Results-Excel.xlsx');
writetable(data, excelFileName);
```
